# Organosolv Lignin Barrier Paper Coatings from Waste Biomass Resources

**DOI:** 10.3390/polym13244443

**Published:** 2021-12-17

**Authors:** Gregor Lavrič, Aleksandra Zamljen, Janja Juhant Grkman, Edita Jasiukaitytė-Grojzdek, Miha Grilc, Blaž Likozar, Diana Gregor-Svetec, Urška Vrabič-Brodnjak

**Affiliations:** 1 Pulp and Paper Institute, Bogišićeva 8, 1000 Ljubljana, Slovenia; gregor.lavric@icp-lj.si (G.L.); janja.juhantgrkman@icp-lj.si (J.J.G.); 2 Department of Catalysis and Chemical Reaction Engineering, National Institute of Chemistry, Hajdrihova 19, 1001 Ljubljana, Slovenia; aleksandra.zamljen@ki.si (A.Z.); edita.jasiukaityte@ki.si (E.J.-G.); miha.grilc@ki.si (M.G.); blaz.likozar@ki.si (B.L.); 3 Faculty of Natural Sciences and Engineering, University of Ljubljana, Snežniška 5, 1000 Ljubljana, Slovenia; diana.gregor@ntf.uni-lj.si

**Keywords:** beech wood biomass, Japanese knotweed invasive species, barrier properties, mechanical properties, molecular weight distribution, nuclear magnetic resonance, size exclusion chromatography

## Abstract

The aim of the study was to isolate lignin from organosolv, beech tree (*Fagus sylvatica*), and Japanese knotweed (*Reynoutria japonica*), to use it for paper surface and to replace part of the non-renewable product resources with bio-based ones. A total of nine coated samples with different lignin formulations and starch were compounded, prepared, and evaluated. The basic (grammage, thickness, specific density), mechanical (elongation at break, tensile, burst and tear indices), and barrier properties (contact angle, water penetration, water vapour permeability, kit test) of the coated papers were investigated. The analysis showed no significant difference in tensile properties between uncoated and coated samples. Furthermore, the decrease in water vapour transmission rate and the lower contact angle for coated samples were nevertheless confirmed. The novel coating materials show promising products with very good barrier properties. Finally, the correlation between structural, morphological, and (other) natural lignin-based factors was revealed, highlighting the importance of parameters such as the equivalence ratio of aliphatic and phenolic hydroxyl groups or the average molecular weight. Tuning functionality by design could optimise performance in the future.

## 1. Introduction

Lignin is the second-most abundant macromolecule group on earth, just after cellulose. It is a ubiquitous biopolymer found in the walls of plant cells, enabling their rigidity and imperviousness [1,2,3,4,5]. Its structure and concentration vary depending on the botanical source, age, and type of cell wall layers. It has a highly branched chemical structure with different functional groups such as hydroxy (OH), methoxy (CH_3_O), carboxyl (COOH), and carbonyl (C=O). The significant variability of lignin types is due to the fact that their chemical structure is highly dependent on the different production streams as well as the source from which they originate. Kraft lignin and lignosulphonate are sulphur-containing lignins. In contrast, organosolv lignin is obtained in the pulping process using low-boiling organic solvents, for instance, ethanol, methanol, and organic acids, without sulphur-containing chemicals [2,3,6]. Such pulping is more ecologically sound in comparison with kraft processes (Azadi et al., 2013). One of the main differences among lignin grades depending on the proportion of functional groups is their water solubility. Lignosulphonate is soluble in water at neutral pH because of its sulphonate groups, whereas kraft lignin is water insoluble at neutral and acidic pH due to its hydrophobic nature [7]. Lignin shows tremendous potential to decrease energy utilisation/pollution and improve biodegradability by replacing synthetic components in different applications [2,6,8,9,10,11]. In paper coatings, lignin is chiefly used to enhance various paper/board properties and replace oil-based components of coatings. For this purpose, it is usually obtained from the black liquor as a by-product in the pulping process. Although large amounts of technical lignins are produced, the immense majority is incinerated rather than used as a precursor for value-added chemicals [2,6]. Examples of its use (especially on laboratory level) to achieve barrier properties of different materials are particularly prominent. Hult et al. [12,13] used modified lignin to develop a paper-based packaging material with barrier properties. The functionalisation enhanced the thermoplastic properties of the lignin, and the coated paperboards showed a significant reduction in water vapour transmission rate (WVTR) and oxygen transmission rate (OTR). This was caused by the non-polar character of some functional groups composing lignin. Narapakdeesakul et al. [14] coated a linerboard using a twin-roll coating unit. The coating consists of lignin (1, 3, 5, 7, and 9%) diluted in a coating medium. Water absorption of linerboards coated with lignin-based coatings was significantly lower than that of uncoated papers, with an optimum absorption for lignin content of 5%. Antonsson et al. [15] used a lignin derivative to coat a filter paper. The lignin derivative coating made the paper surface hydrophobic. It showed a good affinity with lignocellulosic materials, and it was well distributed in the pulp. Hua et al. [16] achieved an enhanced hydrophobicity of paper produced from Kraft pulp resulting in increased water contact angles, and a similar effect was also observed on glass and wood. Combined with the avoidance of volatile organic solvents during the application, their process provided a low environmental footprint solution for the synthesis of hydrophobic coatings. In one of the recent studies in this field, Wang et al. [5] described the lignin redistribution for enhancing barrier properties of cellulose-based materials. The redistribution of alkali lignin on the paper surface showed excellent water, grease, and water vapour barrier. It provided persisted water and grease resistance under long-term moisture or oil direct contact conditions. It also inhibited bacterial growth to a certain degree. Kopačić et al. investigated the use of kraft lignin and lignosulphonate in the surface sizing of paperboard [8]. Their strength properties and hydrophobicity were measured and compared with references as well as with unsized paper. An increase in the strength properties of lignin-sized samples was observed, which was in the same range as references and at least 10% higher compared to unsized paperboard. Moreover, air permeability, hydrophobicity, and water absorptiveness were affected, and depending on the lignin art, an improvement was achieved. Mortarotti in 1987 patented the concept of cardboard treatment with black liquor to increase its strength [17]. The black liquor was added with at least 0.2% of polyvinyl alcohol. In a patent written by Tamminen et al. commercially available softwood lignin was esterified with tall oil fatty acid (TOFA) and tested as barrier material in fibre-based packaging material [18]. The modified lignin samples were applied with a bar coater forming an even coating on the paperboard substrate. A significant decrease in WVTR and OTR value was observed for paperboard coated with the modified lignin as well as pure TOFA used as a reference. In contrast to the TOFA coating, the modified lignin coatings exhibited a high and stable contact angle. The coating material did not affect the tensile strength of the paperboard. Similar improvements (waterproofing and strengthening of the substrate) are also described in the patent of Edye and Tietz from 2014 [19].

The above studies are encouraging for the use of lignin as a coating with water repellent properties without changing the mechanical properties of the cellulosic substrate. However, none of them addressed the organosolv lignin coating from Japanese knotweed. Japanese knotweed was chosen because it is the most invasive plant species in Europe. Various options are being sought to remove it, as it overgrows native plant species. One of the possible solutions is to use it in pulp and paper production. On the other hand, beech wood was used because it is one of the main raw materials in the pulp and paper industry. In order to compare whether the lignin of Japanese knotweed is comparable to the lignin of beech wood in terms of its properties, we conducted the present study.

Our research is the first to present the isolation of lignin from Japanese knotweed, one of the most invasive non-native species worldwide, which was successfully applied to the paper substrate.

## 2. Materials and Methods

One organosolv lignin sample was supplied by Fraunhofer Institute for Interfacial Engineering and Biotechnology (Stuttgart, Germany), whille another two samples were isolated from beech wood and Japanese knotweed in the laboratory (Department of Catalysis and Chemical Reaction Engineering, National Institute of Chemistry, Ljubljana, Slovenia).

### 2.1. Isolation and Analysis of Lignin

Lignin was isolated from beech tree (*Fagus sylvatica*) sawdust and Japanese knotweed (*Reynoutria japonica*) sawdust. A total of 140 g of sawdust and 980 mL of ethanol/water solution in the ratio of 1:1 were placed in the Parr high-pressure reactor (Parr, IL, US). Lignin extraction was induced with the addition of 8.58 mL of 2 M H_2_SO_4_ (Merck, Darmstadt, Germany). The reaction was carried out at 180 °C for 1 h with an agitation speed of 200 rpm. The reactor was pressurised with nitrogen up to 1.7 MPa. The obtained reaction mixture was filtered, and the residue was rinsed with the ethanol/water solution (ethanol was obtained from Merck, Darmstadt, Germany), in the amounts of 670 mL and 170 mL, respectively, and was preheated to 60 °C in order to eliminate trapped lignin from the surface of the solid residue. The residual material was dried at 110 °C for 24 h. Lignin was precipitated by diluting the filtrate four times with distilled water, centrifuged for 15 min at 4500 rpm, washed three times with distilled water, and freeze dried. Taking into account that beech wood contains 24.4% and Japanese knotweed 13.7% of lignin, the yields of isolated lignin were 58.5% and 76.9%, respectively [20,21]

#### Size-Exclusion Chromatography (SEC) and Nuclear Magnetic Resonance (NMR)

Lignin characterisation was carried out using size-exclusion chromatography (SEC) and nuclear magnetic resonance (NMR). Briefly, analysis of the acetylated lignin samples was performed on an HPLC system (Ultimate 3000, Thermo Fisher Scientific, Waltham, MA, US) equipped with a UV detector set at 280 nm and a PLgel 5 μm MIXED D 7.5 × 300 mm column with THF as a mobile phase at a flow rate of 1 cm^3^/min. Polystyrene standards (Polymer Standards Service; PSS, Amherst, US) with molecular weights in the range from 127 kDa to 672 Da were used for calibration [22,23].

For this step, 2D heteronuclear single quantum coherence (HSQC) and quantitative ^31^P NMR spectra were recorded using a Bruker AVANCE NEO 600 MHz NMR (Bruker, Billerica, MA, US) spectrometer equipped with BBFO probe following the protocol reported elsewhere [24,25]. Briefly, 85 mg of each lignin sample was dissolved in 0.6 cm^3^ of DMSO-*d_6_*, which was also an internal chemical shift reference point (δC 39.5; δH 2.35 ppm). Quantitative ^31^P NMR experiments were conducted in CDCl_3_/pyridine 1:1.6 mixture at 25 °C with N-hydroxy-5-norbornene-2,3-dicarboxylic acid imide (Merck, Darmstadt, Germany) (NHND) as an internal standard, precisely following the protocol reported elsewhere [26]. Prior to the analysis, lignin samples were derivatised using 2-chloro-4,4,5,5-tetramethyl-1,2,3-dioxaphospholane (TMDP) (Merck, Darmstadt, Germany).

### 2.2. Coating Solutions

Nine water-based coating solutions were prepared, as shown in Table 1, based on starch and several different lignins. Pure starch Tackidex C062 (Ravago Chemicals UK Ltd., UK) was considered as a reference and was prepared by mixing starch with hot water at 40–45 °C. Coatings with beech organosolv lignin were prepared by adding lignin to the mixture of starch and water. Adjustments of pH values were not undertaken, as they were not crucial for trials and were appropriate for paper coating. Coatings containing Fraunhofer lignin, however, had to be prepared by adding NaOH (Merck, Darmstadt, Germany) due to the acidic character of lignin. The pH of the water was first adjusted to 10.00, and then lignin was added. Next, 5% NaOH was further added until lignin was soluble in water. The mixture was then heated to 40 °C, and starch was added. Since the addition of starch already lowered the pH values of the obtained coatings, further adjustment was unnecessary. The same method was also adopted for the preparation of the additional coatings with beech wood and Japanese knotweed lignin in order to compare the coatings with those containing Fraunhofer lignin. In the case of Fraunhofer lignin and beech lignin coatings, lignin dissolved completely, while Japanese knotweed lignin formed a suspension. The coating solutions were prepared and applied at Pulp and Paper Institute (Ljubljana, Slovenia).

Samples 3 and 4 were the only samples in which the adjusting of pH was not necessary, since they were soluble in water without pH adjustment. The results of mentioned samples and pH are presented also in Table 1.

### 2.3. Surface Coating

The substrate for the surface coating trials was a NiklaTea paper (kindly provided by Papirnica Vevče paper mill, Ljubljana, Slovenia). Paper coating was performed on the laboratory coating machine K Control Coater (RK PrintCoat, Royston, UK). The operating parameters were adjusted in order to achieve the desired application of 5 g/m^2^. The grammage of base paper was 68 g/m^2^; therefore, all grammages of coated papers were about 73 g/m^2^.

### 2.4. Characterisation of Coated Papers

#### 2.4.1. Basic Properties (Grammage, Thickness, Specific Density)

It was necessary to determine grammage and to obtain the amount of coating on all samples, which has an influence on tensile and barrier properties. Grammage was determined in accordance with the ISO 536 standard, where 20 samples of each paper were cut into size 10 × 10 cm and weighed on a scale with a precision of 0.0001 g (Radwag, Radom, Poland). The thickness of samples was measured with a precision digital micrometre (Frank PTI, Birkenau, Germany), to the nearest 0.0001 mm at 10 random locations on each paper. From the grammage and thickness, specific density was calculated, as described in the standard method ISO 534:2011.

#### 2.4.2. Mechanical Properties (Elongation at Break, Burst, Tensile, and Tear Index)

Tensile strength (TS) and elongation at break (E) of papers were determined on a Zwick Roell Z010 (Zwick Roell, Ulm, Germany) tensile testing machine in accordance with ISO 1924-2:2008. The samples were analysed in the standard atmosphere at 23 °C of temperature and 50% of relative humidity. The cross-speed head was 20 mm/min. The distance between the clamps was 18 cm. Paper stripes of 20 cm in length and 1.5 cm in width were used. During the sample stretching, several loads and elongation data per second were recorded until the break of a sample occurred. After the measurement and determination of tensile strength, the tensile index was calculated. The tensile index is tensile strength divided by grammage, and the unit is kN∙m/g.

#### 2.4.3. Burst Index

Burst index [kPa∙m^2^/g] was calculated on the basis of grammage and the results of bursting strength, which was obtained using the Frank burst tester (Frank PTI, Birkenau, Germany) as described in ISO 2758:2014.

#### 2.4.4. Tear Index

Tear index (tear strength divided by grammage expressed in mNm^2/^g) was determined in accordance with ISO 1974:2012, using Lorentzen and Wettre Elmendorf Tear Tester (Lorentzen and Wettre, München, Germany).

#### 2.4.5. Barrier Properties (Contact Angle, Air Permeability, Water Penetration, Water Vapour Transmission Rate, Oil and Grease Resistance)

To analyse the hydrophilic/hydrophobic nature of the paper coatings, barrier properties such as contact angle, water penetration, and water vapour transmission rate were determined.

#### 2.4.6. Contact Angle

The dynamic contact angle was evaluated with the Fibrodat 1100 measuring device (Fibro System AB, Gustafs, Sweden) using distilled water drops of 4 µL in accordance with Tappi T 588.

#### 2.4.7. Water Vapour Transmission Rate (WVTR)

Water vapor transmission rate was determined as described in ISO 2528:2018 standard, at 23 ± 1 °C and 85 ± 2% RH. WVTR values were then calculated as shown in the following equation:

(1) $$WVTR=\frac{\Delta m}{A}\cdot t\left(g/{\mathrm{cm}}^{2}\mathrm{day}\right)$$

where *A* is the tested area in cm^2^, *t* time after 24 h of testing, and Δ*m* is the mass difference of the tested sample.

#### 2.4.8. Air Permeance of Paper

Air permeance of paper was determined as stated in ISO 5636-3:2013, using Frank PTI testing device (Frank PTI, Birkenau, Germany) for the determination of surface roughness and air permeance according to the Bendtsen method.

#### 2.4.9. Oil Resistance

To determine the oil and grease resistance of the coated papers, the standard kit-test was carried out on three replicates from each formula according to the TAPPI T559 standard. Reagents with different rheology and surface energy (numbered from 1 to 12) were dropped onto the paper surface from a height of 13 mm and wiped off after 15 s with a clean cotton swab. The solution with the highest number that did not discolor (darken) the surface was considered to have passed the kit number.

#### 2.4.10. Water Penetration

Water penetration into the structure of the papers was determined using the Emtec PDA.C 02 device (Emtec Electronic, Leipzig, Germany). Within the scope of the method, the material was attached to a support which immersed it in a cell filled with the selected liquid (distilled water in this case). An ultrasonic signal transmitter was mounted on one side of the cell and the receiver on the other. Depending on the properties of the testing material and liquid, the ultrasonic signal was absorbed or scattered, or its intensity was varied. Consequently, a result of the measurement was a change in ultrasonic signal intensity, recorded by the receiver and given as a graph of intensity as a function of time. A programme was selected to determine the penetration of liquids into uncoated/papers. The test was carried out for 60 s with distilled water.

#### 2.4.11. Statistical Analysis

All the properties of the samples were investigated/determined in replicates as described in standards/methods; then, means and standard deviations were calculated and reported.

## 3. Results

### 3.1. Analysis of Lignin

**Figure 1:**
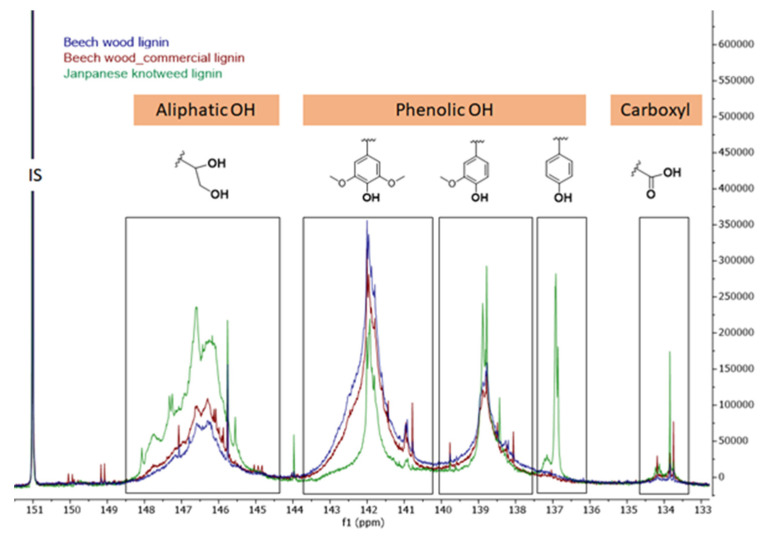
Quantitative ^31^P NMR spectra of lignin samples.

The main structural differences within the lignin samples were disclosed employing the comprehensive lignin characterisation with 2D HSQC, ^31^P NMR, and SEC. Structural characteristics and molecular weights of lignin applied for paper coatings are summarised in Table 2. The reasonable diversity of the samples thus enables the development of structure–properties correlation. For instance, the OH group content, which was evaluated using quantitative ^31^P NMR analysis, demonstrated a significant variation of aliphatic OH groups in the range from 1.06 to 2.96 mmol/g (Table 2, Figure 1). The aliphatic OH/phenolic OH group ratio additionally points to the entirely different reactivity of both in-house isolated organosolv lignin, obviously indicating the effect of the native structure in beech wood and in Japanese knotweed. Furthermore, in contrast to the beech wood lignin and commercial beech wood lignin, remarkably larger lignin fragments (3600 Da) bearing a higher number of β-O-4 linkages (19.5 per 100 C9) were isolated from Japanese knotweed as well. Overall, the effects of such structurally diverse lignin samples were further explored by testing them in paper coatings.

**Figure 2:**
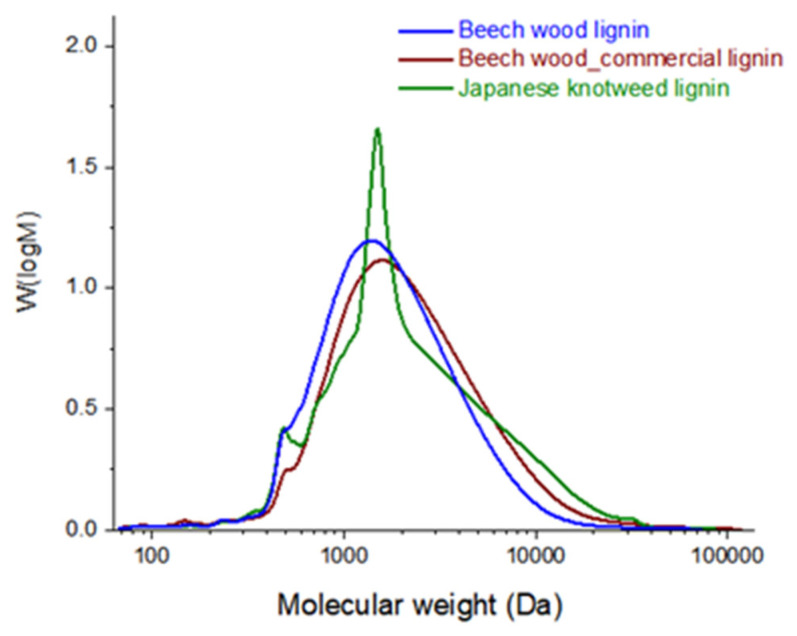
Molecular weight distributions of lignin samples applied in paper coatings.

Molecular weight distributions (MWDs) of the all tested lignins (beech wood lignin, commercial beech wood lignin, and Japanese knotweed) were determined using size-exclusion chromatography (SEC) and are displayed in Figure 2 and listed in Table 2. Analysed lignins, presented in Table 2, were determined to have average Mw 2450 Da, 3350 Da, and 3600 Da, respectively. MWDs, shown in Figure 2, disclose that Japanese knotweed lignin contained a larger part of the high Mw fragments, compared with the other two beechwood lignin and commercial beechwood lignin samples. In addition to that, Japanese knotweed lignin contained a high amount of the monodispersed oligomers with the Mw 1200–2200, which, in combination with the largest molecules, could accordingly affect the coating barrier properties.

### 3.2. Analysis of Coated Papers

Lignin has great potential to increase biodegradability by replacing synthetic components of paper coatings. Mostly, lignin has been used to improve various paper properties and to replace oil-based components in coatings. The results presented in Table 3 show that lignin has a positive effect on barrier properties. Indeed, grammage mainly influences the mechanical properties of the paper. The same applies to the thickness, which causes a difference in the tensile strength, elasticity, bending and tearing stiffness, and water barrier of the paper. About 5 g/m^2^ of the coating was successfully applied to the surface of the samples. The coating with Japanese knotweed lignin (10 and 15%) achieved the highest thickness among the coating samples. This is due to the lumps that were present in the coating.

The tensile strength of paper is dependent on the strength, length, and surface area of fibers, and especially the fiber-to-fiber bonding strength. As it can be seen from Table 3, the base paper and coated papers exhibit similar tensile properties. According to the results, it was found that the coating treatments used did not significantly affect the tensile index of coated papers. In general, the solid fillers can penetrate and remain in the paper structures during the coating process. They can reduce the interaction between the fibres, which leads to a reduction in the tensile strength of the paper [27]. However, the solid fillers used in this study, such as starch and lignin, had large molecular weight, so they were only retained on the surface but could not penetrate into the paper. Therefore, they did not affect the bond between the fibres and did not reduce the tensile index of the coated papers. This was also confirmed by the tear index, which also stayed unchanged or even slightly increased. As tearing resistance is mostly related to inter-fibre bonding, the addition of lignin did not affect the connections between fibres in the paper. Only a slight decrease in burst index, without any statistically significant difference, suggests that the bonding area created between fibres did not change with coating.

It is well known that moisture has a strong influence on the mechanical properties of paper. The water molecules that penetrate a paper structure may become attached to three OH sites in a basic cellulose unit, which leads to a reduction in the internal strength of the paper. Therefore, coatings play an important role in improving the moisture resistance of papers to maintain their strength for their effective use. Thus, in this research, the analysis of the water vapour transmission rate of the coated papers with a desiccant method was performed. The lignin coatings made the paper surface more hydrophobic compared with the uncoated sample (Sample 1), which was confirmed by the WVTR analysis—namely, the ability of a coated mixture of lignin and starch to act as a moisture barrier was investigated by determining WVTR. A more decrease in WVTR was observed for the paper coated with 10% of Japanese knotweed lignin (81.5 g/m^2^∙24 h). Compared with the beech wood lignin, the coating with Japanese knotweed lignin achieved better moisture barrier properties. The outcome can be explicated in terms of the larger lignin fragments, isolated from the Japanese knotweed smoothly covering the coated surface (Table 2, Figure 2).

Furthermore, the contact angle was determined in order to quantitatively measure the wettability of the coating by water. Sufficient wetting between a functional layer and a substrate or between subsequent and underlying functional layers on the substrate is essential for uniform film formation in layered substrates/papers. Functional layer wettability measured by the contact angle method includes test liquids that wet substrates over a period of time, known as the wetting time, and provide insight into the spread or penetration and absorption of fluids into substrates via capillary flow mechanisms [28]. For coated, laminated, or saturated substrates, penetration occurs via a diffusion mechanism. The degree of wetting indicates the interaction between substrates and liquids. A contact angle of less than 90° represents relatively high wettability, while an angle of more than 90° represents lower wettability. The analysis showed that the values of the contact angle water drop were lower at coated samples, compared with uncoated. Decreasing the drop angle of coated papers indicated the spreading behaviour of liquids on the substrate surface. This is due to the water-soluble starch and lignin and the fact that the mentioned components are in direct contact with a larger amount of water–water droplets. The starch granules consist of amylose and amylopectin, which are intensively hydrophilic [14]. Thus, the addition of the starch to the coatings was made for sensitive paper surfaces, which can easily absorb water and then cause a reduction in the contact angle of the coated papers. The results show that coating the paper with starch and organosolv lignin decreased water vapour transmission rate and the contact angle, due to the water solubility of mentioned components. In the applications, water absorption results were considered much more important than contact angle in assessing the water-resistance of papers. A water absorption value could indicate the resistance to the penetration of water molecules through the internal structure of the paper, while the contact angle only indicated the water-resistance of the paper surface.

As expected, the porosity decreased with the amount of coating. It can also be concluded that the starch bound fibres on the surface. The microstructure changed at the coated papers in comparison with porous uncoated paper. The pores were filled with coating solution which prevented the passage of water vapour molecules through the voids between the cellulose fibres. It is obvious that a denser cross-linking structure was created with the aid of coatings, and this contributed to the improved barrier properties of the coated paper, which agrees with the improvement of the WVTR and other properties illustrated above.

Although the oil barrier property is rarely measured, it is often an important aspect in the food and packaging industry. Oil resistance usually results from the relative absence of pores in the paper and is mainly determined by the largest pore size of the paper [29]. The kit test exhibited the maximum value of 11 at samples 3 and 5, coated with 10% beech wood lignin. As expected, the results show that oil permeability decreased with the addition of organosolv lignin in the coating solution. This is also in correlation with the improved barrier properties and a reduction in surface porosity. From the results, it can be concluded that with increasing concentration of the lignin coating, there is a reduction in oil permeability.

**Figure 3:**
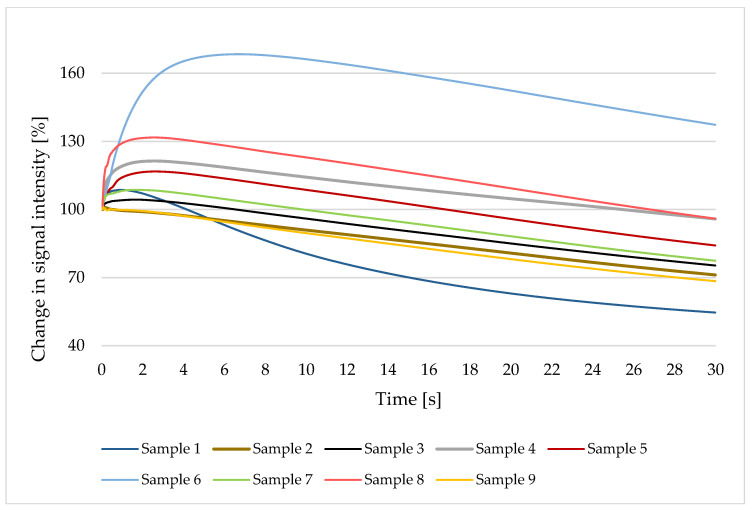
Change in signal intensity (%) during water penetration of analysed papers.

Applied coating solutions filled the voids on the paper surface and partially penetrated into the paper structure. This, among others, affected the way how water penetrated into the paper structure (Figure 3). Air plays an important role in the penetration of liquid into the structure of the uncoated paper, as it is gradually displaced by the penetrating liquid. The process of water penetration into paper can be divided into three stages: immersion, penetration, and soaking. In the first stage, the air on the surface of the paper is replaced by water, and there is no penetration of water into the structure of the paper. At the next stage, i.e., penetration, water begins to penetrate deeper and deeper into the structure of the paper and displaces air. This phase is characterised by a decrease in signal intensity over time; the faster the penetration, the steeper the curve. In the last stage of soaking, the paper is completely soaked with water. Water uptake is complete, and swelling and dissolving may occur. However, the process of water penetration into the structure of coated paper/s is slightly different. In the first step, water wets the coating during immersion and starts to penetrate through the coating layer. In the next step, the paper surface is wetted. The water starts to penetrate inside the paper structure and build up small air bubbles. When the entire coating layer is filled with water, it moves further into paper and builds more and more bubbles. Water-based coatings can start to dissolve during this process, since paper may swell and disintegrate.

As expected, and can be seen from Figure 3, non-coated, base paper reached, on average, the lowest and fastest change in the signal intensity, especially after 6 s, among all samples. This revealed the poorer water penetration of non-coated samples among all the samples analysed.

In a similar way, water penetrated the sample coated only with starch. The addition of lignin in all cases slowed down and limited the soaking with water, thus proving the barrier properties of the paper. This is especially noticeable at samples 6 and 8, in which the penetration of water into the paper structure was about 50% less than in the uncoated sample in the first 30 s after contact with water. The encumbered water permeation could be additionally affected by the reduced amount of the aliphatic OH groups or, more precisely, by the aliphatic OH/phenolic OH ratio in lignin on the coating surface. For instance, the lower the ratio of aliphatic OH/phenolic OH is in the lignin applied for coating, the more hampered liquid penetration is obtained.

Lastly, Figure 3 disclose the direct dependence of the water penetration intensity on the aliphatic OH/phenolic OH ratio, specifically, coatings with beech wood lignin, commercial beech wood lignin, and Japanese knotweed lignin, with the ratios of 0.27, 0.45, 1.23, respectively, showing correspondingly reduced barrier properties for water penetration (samples 6, 8, 10).

## 4. Conclusions

In this study, coatings of beech wood, organosolv lignin from Japanese knotweed, and lignin from beech wood supplied by Fraunhofer were prepared and compared. The prepared coatings were found to have good compatibility with the cellulose paper. The results showed that 10% of each lignin applied as coatings was sufficient for effective moisture and oil barrier behaviour. Indeed, the coated paper with organosolv lignin decreased its water vapour transmission rate and lowered water penetration. An important result is that with the mentioned lignin, promising properties are obtained in the production of coatings, especially when the aim is to produce moisture- and grease-proof paper. The correlation between the lignin structure and the barrier properties of lignin-based coatings was revealed in this work; in particular, the increase in the ratio of aliphatic and phenolic OH groups decreased the intensity of water permeability, while the effect of higher molecular weight lignin in coatings was reflected in the reduced water vapour permeability. Furthermore, the use of lignin-based coatings did not lead to a deterioration in the strength of the coated papers. Overall, the results show promising capabilities of such components that can be applied to the surface of paper substrates and improve their physicochemical properties as well as create a sustainable and water/oil resistant coating. Although we mainly used base paper as an example in this paper, it can be extended to other paper-based materials such as corrugated board, cardboard, etc. This new eco-friendly coating and manufacturing method provides an alternative solution to replace the current petrochemical non-biobased products used for a wide range of packaging applications. At the same time, we introduced the new usability of the raw material obtained from an alternative raw material source.

## Figures and Tables

**Table 1:** Sample names and corresponding surface coating formulations with measured pH (for dissolution of lignin), pH of coating, and viscosity (Brookfield Industries Laboratories Inc., Thomaston, CT, USA).

Sample	Coating Agent	Weight Percentage * (%)	pH Value (Dissolution of Lignin) (25 °C)	pH Value of Coating (25 °C)	Viscosity 100 rpm (mPa∙s)
Sample 1	Basic, non-coated paper	/	/	/	/
Sample 2	Starch	30%	/	7.84	170
Sample 3	Starch + lignin ^B^	30% + 10%	/	7.38	270
Sample 4	Starch + lignin ^B^	30% + 15%	/	7.10	600
Sample 5	Starch + lignin ^B,pH^	30% + 10%	10.27	9.24	220
Sample 6	Starch + lignin ^B,pH^	30% + 15%	9.90	8.95	340
Sample 7	Starch + lignin ^F^	3 0% + 10%	10.60	8.00	145
Sample 8	Starch + lignin ^F^	30% + 15%	10.75	8.70	250
Sample 9	Starch + lignin ^J^	30% + 10%	10.25	8.80	115
Sample 10	Starch + lignin ^J^	30% + 15%	10.70	9.95	275

* relative to water/^B^ Lignin from beech wood/^B,pH^ lignin from beech wood, pH adjustment/^F^ lignin obtained from Fraunhofer/^J^ lignin from Japanese knotweed.

**Table 2:** Structural characteristics and molecular weights of lignin samples determined with NMR and SEC.

Analysis	Characteristic	Units	Beech Wood Lignin	Beech Wood Lignin (Commercial)	Japanese Knotweed Lignin
^31^P NMR	Aliphatic OH	(mmol/g)	1.06	1.51	2.96
	Phenolic OH		3.95	3.36	2.40
	aliphatic OH/phenolic OH		0.27	0.45	1.23
	Total OH		5.06	4.97	5.50
2D HSQC	β-O-4	per 100 C9	0.38	1.87	19.5
	β-5		-	1.6	5.5
	β-β		3.9	6.1	4.6
	S/G		4.1	3.1	0.9
SEC	Mw	Da	2450	3350	3600
	PDI		1.87	2.17	2.42

**Table 3:** Results of basic, tensile, and barrier properties of coated papers (mean values and standard deviation).

Sample	Grammage [g/m^2^]	Thickness [µm]	Specific Density [g/cm^3^]	Burst Index [kPa∙m^2^/g]	Tear Index [mNm^2/^g]	Tensile Index [kN ∙ m/g]	Elongation [%]	Contact Angle [°] (1 s)	Porosity [mL/min]	WVTR at 80% RH [g/m^2^∙24 h]	KIT Test
Sample 1	70.5 ± 0.07	55.3 ± 1.6	1.27	2.64	4.06	2.21	5.87 ± 0.02	89.25 ± 3.43	2.00 ± 0.14	550 (549.9 ± 6.2)	<1
Sample 2	73.7 ± 0.11	60.5 ± 1.4	1.22	2.51	4.56	2.21	5.41 ± 0.16	36.66 ± 2.30	0.00 ± 0.03	150 (151.9 ± 0.15)	8
Sample 3	73.5 ± 0.09	65.7 ± 1.5	1.12	2.50	4.00	2.39	5.67 ± 0.05	37.10 ± 1.53	0.10 ± 0.01	120 (117.6 ± 0.00)	11
Sample 4	74.9 ± 0.13	65.0 ± 1.9	1.15	2.45	3.77	2.41	5.31 ± 0.12	43.01 ± 2.21	0.10 ± 0.02	130 (125 ± 7.4)	8
Sample 5	73.8 ± 0.10	62.7 ± 1.9	1.17	2.57	3.92	2.50	6.19 ± 0.03	32.32 ± 1.09	0.00 ± 0.02	120 (115.6 ± 7.4)	11
Sample 6	73.2 ± 0.11	61.3 ± 1.6	1.14	2.49	3.95	2.26	5.39 ± 0.11	27.19 ± 1.64	0.00 ± 0.03	120 (123.6 ± 2.0)	8
Sample 7	74.1 ± 0.16	63.3 ± 0.8	1.17	2.50	4.11	2.20	5.06 ± 0.09	32.24 ± 2.02	0.05 ± 0.01	140 (137 ± 4.4)	8
Sample 8	74.7 ± 0.09	62.3 ± 1.2	1.20	2.42	4.11	2.26	5.04 ± 0.10	35.71 ± 1.19	0.08 ± 0.02	100 (101.1 ± 8.7)	6
Sample 9	72.5 ± 0.15	66.2 ± 1.5	1.10	2.56	4.74	2.32	6.17 ± 0.01	36.19 ± 2.32	0.05 ± 0.01	80 (81.5 ± 4.1)	8
Sample 10	73.7 ± 0.12	67.8 ± 1.0	1.09	2.53	4.49	2.30	5.85 ± 0.08	34.73 ± 2.62	0.08 ± 0.02	100 (97.0 ± 4.2)	6

## References

[1] Azadi P., Inderwildi O.R., Farnood R., King D.A. (2013). Liquid fuels, hydrogen and chemicals from lignin: A critical review. Renew. Sustain. Energy Rev..

[2] Liao J.J., Latif N.H.A., Trache D., Brosse N., Hussin M.H. (2020). Current advancement on the isolation, characterization and application of lignin. Int. J. Biol. Macromol..

[3] Mansouri N.E., El Salvadó J. (2006). Structural characterization of technical lignins for the production of adhesives: Application to lignosulfonate, kraft, soda-anthraquinone, organosolv and ethanol process lignins. Ind. Crops Prod..

[4] Singh R., Shukla A., Tiwari S., Srivastava M. (2014). A review on delignification of lignocellulosic biomass for enhancement of ethanol production potential. Renew. Sustain. Energy Rev..

[5] Wang W., Guo T., Sun K., Jin Y., Gu F., Xiao H. (2019). Lignin Redistribution for Enhancing Barrier Properties of Cellulose-Based Materials. Polymers.

[6] Dessbesell L., Paleologou M., Leitch M., Pulkki R., Xu C. (2020). Global lignin supply overview and kraft lignin potential as an alternative for petroleum-based polymers. Renew. Sustain. Energy Rev..

[7] Javed A., Ullsten H., Rättö P., Järnström L. (2018). Lignin-containing coatings for packaging materials. Nord. Pulp Paper Res. J..

[8] Kopacic S., Ortner A., Guebitz G., Kraschitzer T., Leitner J., Bauer W. (2018). Technical lignins and their utilization in the surface sizing of paperboard. Ind. Eng. Chem. Res..

[9] Maximova N., Laine J., Stenius P. (2005). Adsorption of lignin-cationic starch complexes on cellulose fibres and their effect on sheet properties. Pap. Ja Puu/Pap. Timber.

[10] Altay B.N., Aksoy B., Banerjee D., Maddipatla D., Fleming P.D., Bolduc M., Cloutier S.G., Atashbar M.Z., Gupta R.B., Demir M. (2021). Lignin-Derived Carbon-Coated Functional Paper for Printed Electronics. ACS Appl. Electron. Mater..

[11] Gumowska A., Kowaluk G., Labidi J., Robles E. (2019). Barrier properties of cellulose nanofiber film as an external layer of particleboard. Clean Technol. Environ. Policy.

[12] Hult E.L., Koivu K., Asikkala J., Ropponen J., Wrigstedt P., Sipilä J., Poppius-Levlin K. (2013). Esterified lignin coating as water vapor and oxygen barrier for fiber-based packaging. Holzforschung.

[13] Hult E.L., Ropponen J., Poppius-Levlin K., Ohra-Aho T., Tamminen T. (2013). Enhancing the barrier properties of paper board by a novel lignin coating. Ind. Crops Prod..

[14] Narapakdeesakul D., Sridach W., Wittaya T. (2013). Recovery characteristics and potential use as linerboard coatings material of lignin from oil palm empty fruit bunches black liquor. Ind. Crops Prod..

[15] Antonsson S., Henriksson G., Johansson M., Lindström M.E. (2008). Low Mw-lignin fractions together with vegetable oils as available oligomers for novel paper-coating applications as hydrophobic barrier. Ind. Crops Prod..

[16] Hua Q., Liu L.-Y., Karaaslan M.A., Renneckar S. (2019). Aqueous Dispersions of Esterified Lignin Particles for Hydrophobic Coatings. Front. Chem..

[17] Mortarotti E. (1987). A process for the manufacturing of paper, particulary corrugated paperboard, and resulting product. European Patent.

[18] Tamminem T., Ropponen J., Hult E.-L., Puppius-Levlin K. (2018). Functionalized lignin and method of producing the same. European Patent.

[19] Edye L.A., Tietz A.J. (2020). U.S. Patent.

[20] Jasiukaitytė-Grojzdek E., Huš M., Grilc M., Likozar B. (2020). Acid-catalysed α-O-4 aryl-ether bond cleavage in methanol/(aqueous) ethanol: Understanding depolymerisation of a lignin model compound during organosolv pretreatment. Sci. Rep..

[21] Lecerf A., Patfield D., Boiché A., Riipinen M.P., Chauvet E., Dobson M. (2007). Stream ecosystems respond to riparian invasion by Japanese knotweed (*Fallopia Japonica*). Can. J. Fish. Aquat..

[22] Jasiukaitytė-Grojzdek E., Kunaver M., Crestini C. (2012). Lignin structural changes during liquefaction in acidified ethylene glycol. J. Wood Chem. Technol..

[23] Jasiukaitytė-Grojzdek E., Vicente F.A., Grilc M., Likozar B. (2021). Ambient-pressured acid-catalysed ethylene glycol organosolv process: Liquefaction structure–Activity relationships from model cellulose–Lignin mixtures to lignocellulosic wood biomass. Polymers.

[24] Tran F., Lancefield C.S., Kamer P.C.J., Lebl T., Westwood N.J. (2015). Selective modification of the β-β linkage in DDQ-treated Kraft lignin Analysed by 2D NMR spectroscopy. Green Chem..

[25] Zijlstra D.S., de Santi A., Oldenburger B., de Vries J., Barta K., Deuss P.J. (2019). Extraction of lignin with high β-O-4 content by mild ethanol extraction and its effect on the depolymerization yield. J. Vis. Exp..

[26] Meng X., Crestini C., Ben H., Hao N., Pu Y., Ragauskas A.J., Argyropoulos D.S. (2019). Determination of hydroxyl groups in biorefinery resources via quantitative ^31^P NMR Spectroscopy. Nat. Protoc..

[27] Han J.H., Krochta J.M. (2001). Physical properties and oil absorption of whey-protein-coated paper. J. Food Sci..

[28] Sarah K., Ulrich H. (2018). Short timescale wetting and penetration on porous sheets measured with ultrasound, direct absorption and contact angle. RSC Adv..

[29] Song Z., Xiao H., Li Y. (2015). Effects of renewable materials coatings on oil resistant properties of paper. Nord. Pulp Paper Res. J..

